# A set of stage-specific gene transcripts identified in EK stage X and HH stage 3 chick embryos

**DOI:** 10.1186/1471-213X-7-60

**Published:** 2007-06-01

**Authors:** Bo Ram Lee, Heebal Kim, Tae Sub Park, Sunjin Moon, Seoae Cho, Taesung Park, Jeong Mook Lim, Jae Yong Han

**Affiliations:** 1 Department of Food and Animal Biotechnology, Seoul National University, Seoul 151-921, Korea; 2 Avicore Biotechnology Institute Inc., Hanlim Human Tower #707, Gyeonggi-Do 435-050, Korea; 3 Department of Statistics, Seoul National University, Seoul 151-747, Korea

## Abstract

**Background:**

The embryonic developmental process in avian species is quite different from that in mammals. The first cleavage begins 4 h after fertilization, but the first differentiation does not occur until laying of the egg (Eyal-Giladi and Kochav (EK) stage X). After 12 to 13 h of incubation (Hamburger and Hamilton (HH) stage 3), the three germ layers form and germ cell segregation in the early chick embryo are completed. Thus, to identify genes associated with early embryonic development, we compared transcript expression patterns between undifferentiated (stage X) and differentiated (HH stage 3) embryos.

**Results:**

Microarray analysis primarily showed 40 genes indicating the significant changes in expression levels between stage X and HH stage 3, and 80% of the genes (32/40) were differentially expressed with more than a twofold change. Among those, 72% (23/32) were relatively up-regulated at stage X compared to HH stage 3, while 28% (9/32) were relatively up-regulated at HH stage 3 compared to stage X. Verification and gene expression profiling of these GeneChip expression data were performed using quantitative RT-PCR for 32 genes at developmental four points; stage X (0 h), HH stage 3 (12 h), HH stage 6 (24 h), and HH stage 9 (30 h). Additionally, we further analyzed four genes with less than twofold expression increase at HH stage 3. As a result, we identified a set of stage-specific genes during the early chick embryo development; 21 genes were relatively up-regulated in the stage X embryo and 12 genes were relatively up-regulated in the HH stage 3 embryo based on both results of microarray and quantitative RT-PCR.

**Conclusion:**

We identified a set of genes with stage-specific expression from microarray Genechip and quantitative RT-PCR. Discovering stage-specific genes will aid in uncovering the molecular mechanisms involved the formation of the three germ layers and germ cell segregation in the early chick embryos.

## Background

In embryogenesis, a series of developmental events begins immediately after fertilization. During the early embryo development, the expression of many genes is spatiotemporally triggered or suppressed, under tight transcriptional control. However, the intricate changes in gene expression in the early embryo have yet to be investigated in detail in mammals or birds.

As avian species are oviparous, the embryo is readily accessible even at the earliest stages and can effectively be manipulated for purposes including profiling the genes expressed in embryogenesis. Recently, the chicken genome was completely sequenced [1], and furthermore, Burt *et al *[2] reported that the organization of the human genome is actually closer to that of the chicken than the mouse. Thus, the chicken is an interesting and relevant experimental animal model.

In avian species, the embryonic developmental process is quite different from that in mammals. The first cleavage begins 4 h after fertilization, as the embryos enter the magnum of the reproductive duct [3], but the first differentiation does not occur until the egg is laid. Cells in the embryo continue to proliferate until the Eyal-Giladi and Kochav stage X and the laid egg consists of 40,000 to 60,000 undifferentiated embryonic cells [4]. After 12 to 13 h of incubation at Hamburger and Hamilton (HH) stage 3, the primitive streak extends to about the center of the area pellucida [5]. The groove in the primitive streak is gradually established as the cells of surrounding epiblast rapidly divide and migrate to the lower regions of the embryo, where they spread laterally across the surface of the yolk into two layers, the endoderm and mesoderm.

Together with when the three germ layers begin to form, the first appearance of germ cells is an important event during the early embryo development. Primordial germ cells, the progenitors of sperm or egg cells after sexual maturity, first appear from the epiblast in the blastoderm at stage X and translocate to the hypoblast of the area pellucida [6-8]. During gastrulation, they circulate through the vascular system and finally settle in the gonadal anlagen [9].

Understanding the molecular mechanisms that underlie germ cell segregation during early embryogenesis is important not only from the perspective of fundamental research in embryology but also from that of practical use of genetic methods. Additionally, discovering developmental stage-specific genes will aid in uncovering the molecular mechanism involved in the early stages of chicken embryo development.

In this study, we investigated the linkage of gene expression with morphological events, including germ cell segregation and identified gene transcripts from microarray GeneChip technology (stage X versus HH stage 3). We further analyzed our gene expression data using quantitative RT-PCR.

## Results

### Microarray data analysis

**Figure 1:**
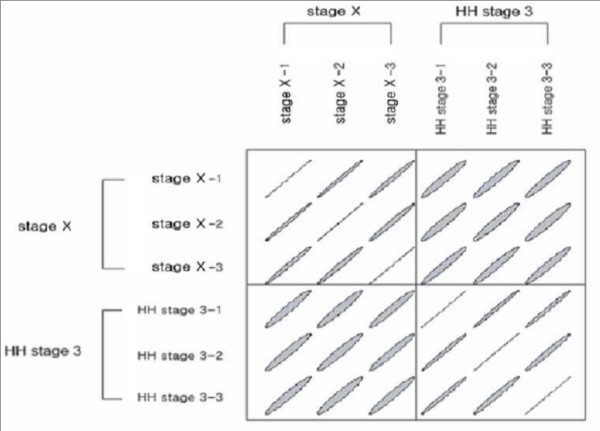
**Plot of correlation matrix between all pairs**. The ellipse represents a level curve of the density of a bivariate normal with the matching correlation that shows the group variances are higher than the within variances. Each sample was performed in triplicate.

**Figure 2:**
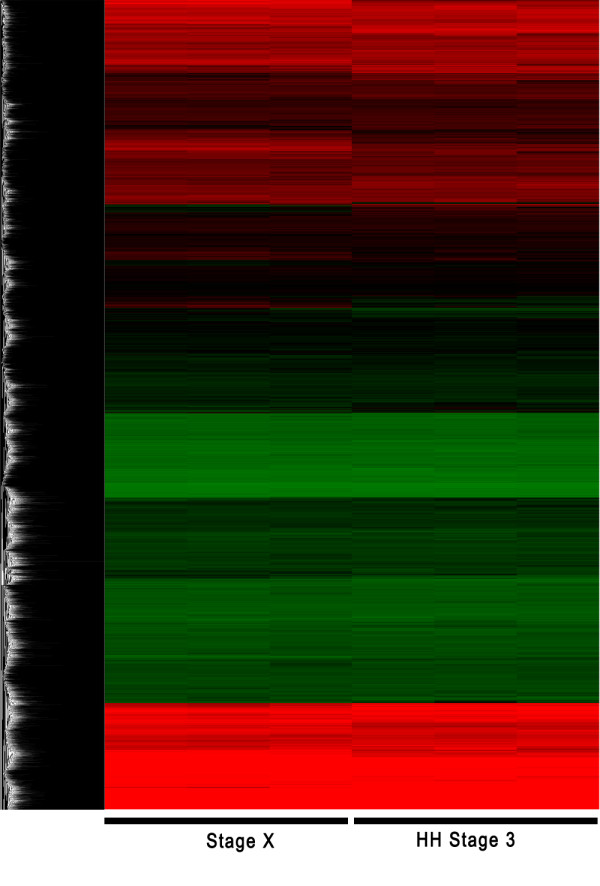
**Clustering result of the microarray experiment**. Clustering is conducted from the normalized expression data of all probes. Each row represents the relative gene expression of a single gene. Each column represents a sample. High expression relative to mean are colored red, and low expression relative to mean are colored green, and Black represents mean expression levels of the experiment. The clustering result is displayed by using TreeView software.

Microarray analyses were conducted to identify genes that were differentially expressed between Eyal-Giladi and Kochav stage X and Hamburger and Hamilton (HH) stages 3 embryos. Raw expression levels were corrected and normalized using the RMA function and the affy software. A correlation matrix showed that the between-group variances were higher than the within-group variances (Figure 1). Hierarchical clustering was conducted upon all probes by using Cluster, and the clustering result was displayed using TreeView software (Figure 2).

Microarray analysis primarily showed 40 genes indicating the significant changes in expression levels between stage X and stage 3; 27 were up-regulated in the stage X embryo (Table 1) and 13 were up-regulated in the HH stage 3 embryo (Table 2). 80% (32/40) of these genes were differentially expressed with more than a twofold change. Among those, 72% (23/32) were relatively up-regulated at stage X compared to HH stage 3, while 28% (9/32) were relatively up-regulated at HH stage 3 compared to stage X. To verify and further characterize the GeneChip expression data, quantitative RT-PCR was performed.

**Table 1:** List of the up-regulated genes in stage X embryo from microarray data

#	GeneChip Probe Set ID	Gene Title	GO Molecular Function Description^a^	log_2 _FC^b^
1	Gga.11040.1.S1_at	similar to hypothetical protein BC009518	NA	1.208
2	Gga.12503.2.S1_at	Nucleoredoxin	electron carrier activity	1.874
3	Gga.13029.1.S1_at	Finished cDNA, clone ChEST43l14	NA	1.561
4	Gga.14754.1.S1_at	death associated transcription factor 1	cation binding, metal ion binding	1.514
5	Gga.16489.1.S1_at	Finished cDNA, clone ChEST769a21	NA	1.111
6	Gga.17114.2.S1_a_at	Finished cDNA, clone ChEST485a9	NA	1.675
7	Gga.8833.1.S1_at	Transcribed locus	NA	2.036
8	GgaAffx.10043.1.S1_at	similar to Topoisomerase I binding, arginine/serine-rich	Catalytic activity	1.498
9	GgaAffx.11175.1.S1_at	similar to 12-transmembrane domain co-transporter Ce11	Catalytic activity	1.561
10	GgaAffx.11653.1.S1_s_at	serine/threonine kinase 17b (apoptosis-inducing)	purine nucleotide binding, transferase activity, transferring phosphorus-containing groups	1.795
11	GgaAffx.21832.1.S1_s_at	fibroblast growth factor 13	Growth factor activity	1.822
12	GgaAffx.2367.1.S1_at	NA	NA	1.775
13	GgaAffx.4101.1.S1_at	similar to transmembrane protein 20	NA	1.600
14	GgaAffx.4973.2.S1_s_at	similar to TNF receptor-associated factor 6	protein binding/zinc ion binding	2.174
15	GgaAffx.5963.1.S1_at	similar to RIKEN cDNA C030048B08	nucleotide binding	1.072
16	GgaAffx.8887.1.S1_s_at	similar to VANIN 3	hydrolase activity, acting on carbon-nitrogen (but not peptide) bonds	2.237
17	GgaAffx.9105.1.S1_at	similar to Stromal interaction molecule 2 precursor	catalytic activity/scavenger receptor activity	1.649
18	GgaAffx.9296.3.S1_s_at	similar to RNA polymerase II elongation factor ELL2	catalytic activity	1.404
19	Gga.17330.1.S1_at	splicing factor, arginine/serine-rich 15	nucleotide binding/nucleic acid binding	2.583
20	Gga.318.1.S1_at	potassium intermediate/small conductance calcium-activated channel, subfamily N, member 2	alpha-type channel activity, calmodulin binding, cation transporter activity, ion channel activity	2.368
21	Gga.4401.1.S1_a_at	prostaglandin-endoperoxide synthase 2	cation binding, dioxygenase activity, metal ion binding, oxidoreductase activity, acting on peroxide as acceptor	3.314
22	GgaAffx.20623.1.S1_s_at	LOC420789	NA	2.979
23	GgaAffx.7728.1.S1_at	similar to velo1	NA	2.594
24	GgaAffx.10088.2.A1_at	similar to hypothetical protein MGC46520	serine-type endopeptidase activity	0.199
25	Gga.9919.1.S1_at	similar to pre-mRNA splicing factor SF3b 155 kDa subunit	Catalytic activity	0.458
26	Gga.550.1.S1_at	gamma-aminobutyric acid (GABA) A receptor, alpha 6	alpha-type channel activity, ion channel activity, neurotransmitter receptor activity, transmembrane receptor activity	0.513
27	GgaAffx.6386.1.S1_at	similar to Ubiquitin ligase protein RNF8	protein binding/zinc ion binding	0.835

^a^Gene transcripts were classified using NetAffx analysis tool provided by the Affymetrix.

^b^Log_2 _fold changes calculated from the microarray data (right column) are indicated. Log_2_FC > 1 indicates that gene expression at stage X was up-regulated more than twice than that at HH stage 3. NA is not annotated from NetAffx analysis tool.

**Table 2:** List of the up-regulated genes in HH stage 3 embryo from microarray data

#	GeneChip Probe Set ID	Gene Title	GO Molecular Function Description^a^	log_2 _FC^b^
1	GgaAffx.21413.1.S1_s_at	Finished cDNA, clone ChEST629c13	NA	-2.001
2	Gga.7813.1.S1_at	Finished cDNA, clone ChEST148d1	NA	-1.592
3	Gga.8314.1.S1_at	Transcribed locus	NA	-1.523
4	Gga.12166.1.S1_at	zinc finger homeobox 1b	nucleic acid binding/transcription factor activity/catalytic activity/zinc ion binding/sequence-specific DNA binding	-1.474
5	Gga.19405.1.S1_at	Finished cDNA, clone ChEST666e20	NA	-1.318
6	GgaAffx.9643.1.S1_at	proprotein convertase PC6	subtilase activity	-1.285
7	Gga.16092.1.S1_at	Finished cDNA, clone ChEST357i21	NA	-1.203
8	GgaAffx.21109.1.S1_s_at	Finished cDNA, clone ChEST172i5	guanyl-nucleotide exchange factor activity	-1.084
9	Gga.13785.1.S1_at	heterogeneous nuclear ribonucleoprotein L-like	NA	-1.082
10	Gga.18924.1.S1_at	Finished cDNA, clone ChEST628k5	NA	-0.973
11	Gga.15987.1.S1_at	Finished cDNA, clone ChEST481o6	NA	-0.662
12	GgaAffx.11516.1.S1_s_at	similar to septin 2	nucleotide binding/GTP binding/ATP binding	-0.650
13	Gga.12446.1.S1_s_at	staufen, RNA binding protein (Drosophila)	double-stranded RNA binding	-0.391

^a^Gene transcripts were classified using NetAffx analysis tool provided by the Affymetrix.

^b^Log_2 _fold changes calculated from the microarray data (right column) are indicated. Log_2_FC < -1 indicates that gene expression at stage X was down-regulated more than twice than that at HH stage 3. NA is not annotated from NetAffx analysis tool.

### Confirmation of differentially expressed genes via quantitative RT-PCR

**Figure 3:**
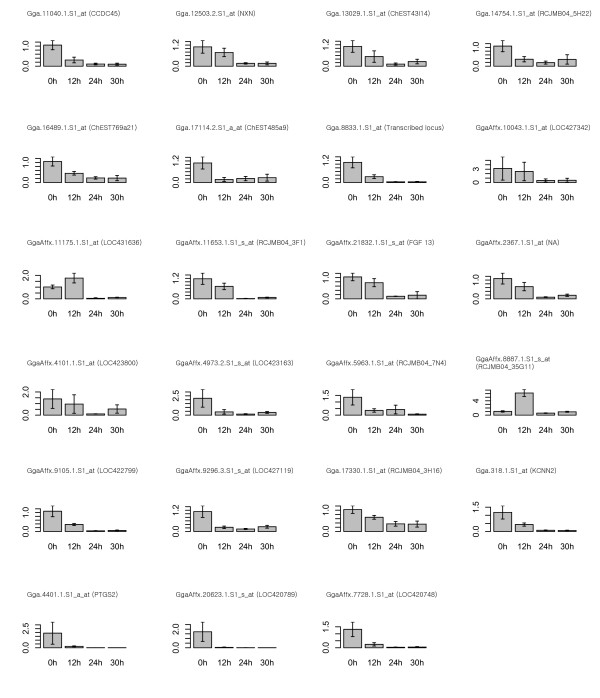
**Quantification of the relative expression of the 23 genes selected as being up-regulated in stage X embryo compared to Hamburg and Hamilton (HH) stage 3**. Quantitative RT-PCR was conducted at stage X (0 h), HH stage 3 (12 h), HH stage 6 (24 h), and HH stage 9 (30 h) embryos. The relative quantification of gene expression was analyzed by the 2^-ΔΔCt ^method. NA is not annotated from NetAffx analysis tool.

**Figure 4:**
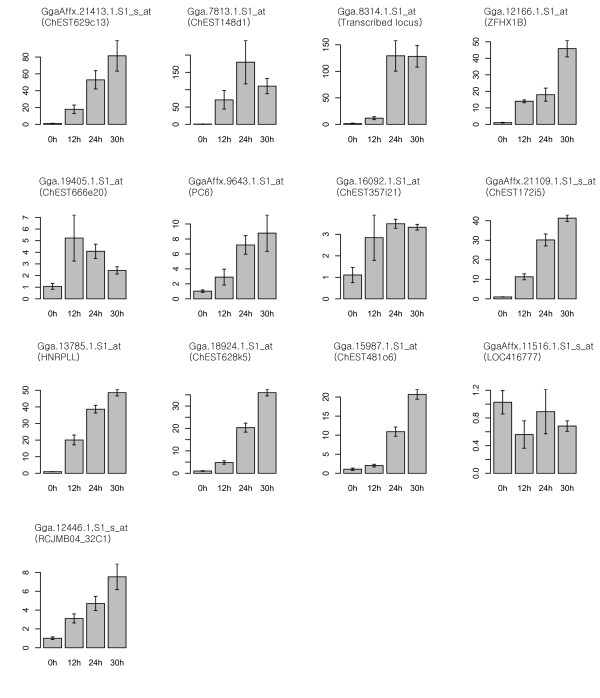
**Quantification of the relative expression of the 13 genes selected as being up-regulated in Hamburg and Hamilton (HH) stage 3 embryo compared to stage X**. Quantitative RT-PCR was conducted at stage X (0 h), HH stage 3 (12 h), HH stage 6 (24 h), HH stage 9 (30 h) embryos. The relative quantification of gene expression was analyzed by the 2^-ΔΔCt ^method.

**Figure 5:**
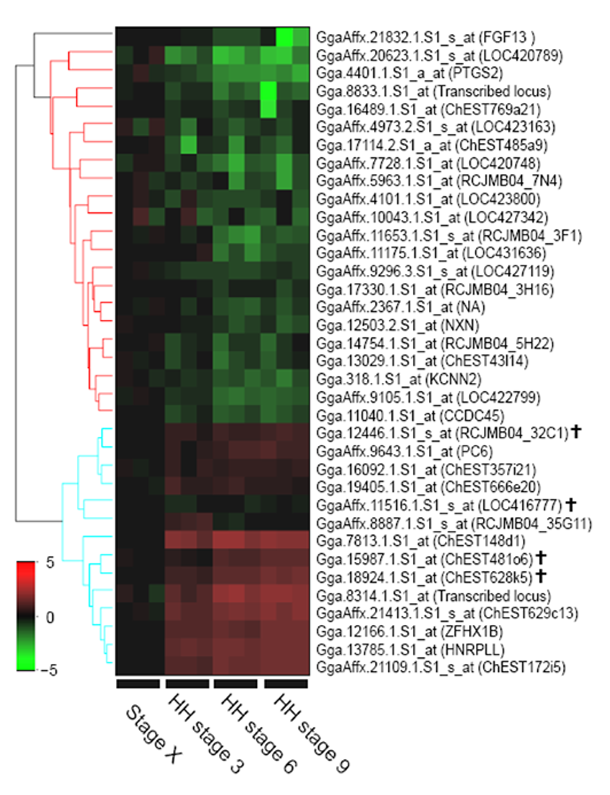
**Hierarchical clustering of differentially expressed genes analyzed by quantitative RT-PCR**. Based on the microarray analysis, the 23 genes selected as being up-regulated at stage X and the 13 genes selected as being up-regulated at HH stage 3 were confirmed by quantitative RT-PCR at stage X (0 h), HH stage 3 (12 h), HH stage 6 (24 h), and HH stage 9 (30 h). Most (91.7%, 33/36) of genes were consistent with results as shown of microarray data analysis. Color indicates the relative expression levels of each gene, with red indicating higher expression, green indicating negative expression. Black represents a control group that is normalized for the relative quantification of gene expression. Four genes with less than twofold expression increase at HH stage 3 were indicated (≸). NA is not annotated from NetAffx analysis tool.

The microarray gene expression data were confirmed using quantitative RT- PCR for 32 genes showing the more than twofold changes in expression levels between stage X and HH stage 3. Additionally, we further analyzed 4 genes with less than twofold increase in expression at HH stage 3 compared to stage X. Although cut-off was less than twofold, these extra genes might be important in morphological events including germ cell segregation. Thus, expression profiles of total 36 gene transcripts (23 and 13 at stage X and HH stage 3, respectively) were quantified by RT-PCR (Table 3, 4). Furthermore, to investigate expression patterns of those transcripts during the early chick embryo development, we extended embryo development stages from stage X to HH stage 9 and prepared total mRNAs from 4 developmental points for quantitative RT-PCR; stage X (0 h), HH stage 3 (12 h), HH stage 6 (24 h), and HH stage 9 (30 h). Most (91.7%, 33/36) of gene transcripts were consistent with results as shown in microarray data analysis between stage X and HH stage 3. 21 genes were relatively up-regulated in the stage X embryo (Figure 3) and 12 genes were relatively up-regulated in the HH stage 3 embryo (Figure 4). However, 2 genes (GgaAffx.11175.1.S1_at and GgaAffx.8887.1.S1_s_at) at stage X and 1 gene (GgaAffx.11516.1.S1_s_at) at HH stage 3 showed the opposite results in quantitative PCR analysis compared to microarray data. The relative gene expression profiles of stage-specific genes were also confirmed from stage X (0 h) to HH stage 9 (30 h) (Figure 3, 4). Based on the quantification RT-PCR results, Hierarchical clustering was performed (Figure 5).

**Table 3:** List of the primer pairs for quantitative RT-PCR. Twenty-three genes were selected as being up-regulated in stage X embryo from microarray data

#	GeneChip Probe Set ID	Gene Title	Forward	Reverse
1	Gga.11040.1.S1_at	similar to hypothetical protein BC009518	tgctggtcaacttatgccac	gcactggaactgaaatgctg
2	Gga.12503.2.S1_at	Nucleoredoxin	gcagaacaaggtggtggg	gtggtcggaggagatgaaga
3	Gga.13029.1.S1_at	Finished cDNA, clone ChEST43l14	tgcccttcatacctcctgac	catttattcttccaccccca
4	Gga.14754.1.S1_at	death associated transcription factor 1	gacaaagcagcagagcacac	gcacccaaactggaagatg
5	Gga.16489.1.S1_at	Finished cDNA, clone ChEST769a21	gaatcggtgtgtgacttggtt	tctccaacttccacagcaca
6	Gga.17114.2.S1_a_at	Finished cDNA, clone ChEST485a9	gtttcccttcccaacaggag	aaaagcccctctgattcca
7	Gga.8833.1.S1_at	Transcribed locus	agggcggctttttcagatac	ttgctttttgctctcctcatc
8	GgaAffx.10043.1.S1_at	similar to Topoisomerase I binding, arginine/serine-rich	catcttccttcctggggact	ctgcctgtgcttcttgactg
9	GgaAffx.11175.1.S1_at	similar to 12-transmembrane domain co-transporter Ce11	agccttcagagagcagcaac	ttggagaacacaaagacccc
10	GgaAffx.11653.1.S1_s_at	serine/threonine kinase 17b (apoptosis-inducing)	atgcttgaacagaaaacggg	ttgaacttagaaagggggca
11	GgaAffx.21832.1.S1_s_at	fibroblast growth factor 13	acagcaggcaaggataccac	aagacccaaataccaacccc
12	GgaAffx.2367.1.S1_at	NA	gaacctgtaaccccgaatga	ctttccttttccgatttccc
13	GgaAffx.4101.1.S1_at	similar to transmembrane protein 20	tgattctcctctactatgctttcca	tcccaaacgctgtatttttctt
14	GgaAffx.4973.2.S1_s_at	similar to TNF receptor-associated factor 6	atttgagcctgccctcttct	tgtgagtgttttgcgtctcc
15	GgaAffx.5963.1.S1_at	similar to RIKEN cDNA C030048B08	tccaagaaaccagggagaag	tgactgtgattgccagaagg
16	GgaAffx.8887.1.S1_s_at	similar to VANIN 3	gaagggaaactggtggctc	cagaaagggcaagacattca
17	GgaAffx.9105.1.S1_at	similar to Stromal interaction molecule 2 precursor	gggtagttatgccccgagtt	ccagtgtgcttggaggtttt
18	GgaAffx.9296.3.S1_s_at	similar to RNA polymerase II elongation factor ELL2	accaccagcaccagtcattt	gcttcttgttcatcagggga
19	Gga.17330.1.S1_at	splicing factor, arginine/serine-rich 15	ggagccctctcaagtcactg	tgaccggagactgagctttt
20	Gga.318.1.S1_at	potassium intermediate/small conductance calcium-activated channel, subfamily N, member 2	catcttgctcggactcatca	gcgtaagaaaaggcaagtcg
21	Gga.4401.1.S1_a_at	prostaglandin-endoperoxide synthase 2	tgcaacgatatggctgagag	gggtgccagtggtacagagt
22	GgaAffx.20623.1.S1_s_at	LOC420789	tgttgaagcatacttccctt	cttgcctcttcatttgattc
23	GgaAffx.7728.1.S1_at	similar to velo1	accagtcccagagaacatgg	gtgattttgcccgttgactt

NA is not annotated from NetAffx analysis tool.

**Table 4:** List of the primer pairs for quantitative RT-PCR.  Thirteen genes were selected as being up-regulated in Hamburg and Hamilton (HH) stage 3 embryo from microarray data.

#	GeneChip Probe Set ID	Gene Title	Forward	Reverse
1	GgaAffx.21413.1.S1_s_at	Finished cDNA, clone ChEST629c13	cccagtcccagtgtttgagt	acaagcgatgaaagaaagcc
2	Gga.7813.1.S1_at	Finished cDNA, clone ChEST148d1	cagcacacagaaatgggaga	ctgaagaagcacacagcagg
3	Gga.8314.1.S1_at	Transcribed locus	tgtcataaacggggaaaagtg	gctgaaagagactgtgataaaccatac
4	Gga.12166.1.S1_at	zinc finger homeobox 1b	aagtcgagtgcaattcgtaca	tggactaaaggggcagttca
5	Gga.19405.1.S1_at	Finished cDNA, clone ChEST666e20	tgctctgtccttcttctgcc	ccccaaccatctctccagt
6	GgaAffx.9643.1.S1_at	proprotein convertase PC6	tcttcttcaactgcctgcct	gaacaccgtccttcgtcact
7	Gga.16092.1.S1_at	Finished cDNA, clone ChEST357i21	ttggcaggaaggaagacaag	gcaactctacgaactggctg
8	GgaAffx.21109.1.S1_s_at	Finished cDNA, clone ChEST172i5	ggttggaatcttgtggcagt	gcttgttggtcggtcttctc
9	Gga.13785.1.S1_at	heterogeneous nuclear ribonucleoprotein L-like	gctttcatcaggttaatgttgc	catgctaatgcaaacggaaa
10	Gga.18924.1.S1_at	Finished cDNA, clone ChEST628k5	accacaggcagtacggctac	ccccaaggaaaatttaaaacg
11	Gga.15987.1.S1_at	Finished cDNA, clone ChEST481o6	tcataccagtctcaagcaggg	tccttcattcctcactcaaaca
12	GgaAffx.11516.1.S1_s_at	similar to septin 2	acttccgttcagagaggctg	agcacccgtccacttagaga
13	Gga.12446.1.S1_s_at	staufen, RNA binding protein (Drosophila)	ccgcagattgattttccttg	acttgcaaggctatcgtgct

## Discussion

Recently, with chicken genome data and advances in DNA microarray technology, integration of these genomic data with the wealth of existing descriptive embryology will strengthen the position of the chicken embryo as a useful experimental model [10-13]. The chicken embryo provides an excellent model system for studying the developmental biology of vertebrates because of the accessibility of the chick embryo, allowing embryonic manipulations that are not possible in a mammalian system. But, there are few reports on microarray GeneChip technology and functional genomics for profiling gene transcripts expressed during early embryo development in avian species.

To investigate the linkage of gene expression with morphological events, including germ cell segregation, we examined differentially expressed genes between undifferentiated Eyal-Giladi and Kochav (EK) stage X and differentiated Hamburger and Hamilton (HH) stage 3 chicken embryos. Specifically, we used microarray GeneChip technology to identify a set of genes with stage-specific expression, and its results were confirmed by the relative gene expression profiling using quantitative RT-PCR. From our results based on both of microarray and quantitative RT-PCR, we identified a set of stage-specific genes in the developing chick embryo: 21 genes were relatively up-regulated in stage X embryo and 12 genes were relatively up-regulated in the HH stage 3 embryo.

Confirming GeneChip results with quantitative RT-PCR is important in determining whether a specific transcript is truly up- or down-regulated. Our quantitative RT-PCR results demonstrated a higher than 90% concordance with the GeneChip data, consistent with the findings of Rajeevan *et al*. [14]. Their results showed a similar the degree of agreement between GeneChip and QRT-PCR and that the agreement was related to hybridization intensity, with the highest level of concordance for those genes with the highest signal intensity on the GeneChip. From the quantitative RT-PCR in this study, most (21/23) of genes up-regulated in stage X embryo gradually decreased according to developmental process, while almost (12/13) of genes up-regulated in HH stage 3 embryo were relatively higher than in stage X and increased according to ages of incubation time until HH stage 9 (30 h). However, three gene transcripts (2 at stage X and 1 at HH stage 3) showed the discrepancy between microarray data and the quantitative RT-PCR analysis. Thus, further intensive experiments such as Northern blot and *in situ *hybridization as well as functional study would be conducted.

The freshly laid egg, at stage X, consists of 40,000 to 60,000 undifferentiated embryonic cells. At this stage, notably, anti-oxidation-related transcripts such as Gga.12503.2.S1_at (*Nucleoredoxin*) and Gga.4401.1.S1_a_at (*prostaglandin-endoperoxide synthase 2*) were expressed higher than at HH stage 3. Oxidative stress is one of the main damage sources in living organisms, and the anti-oxidant defence mechanisms of biological systems play major roles in preventing oxidative stress and the consequent cell damage [15]. Thus, higher expression of anti-oxidation-related transcripts could be one of the critical mechanisms for survival at stage X embryo.

Signal transduction pathways are known to play key roles in cell fate commitments during early embryogenesis. *FGF13*, highly expressed in stage X embryo, would be involved in the signalling transduction during the early chick embryogenesis. FGF signaling not only mediates cell fate specification in the early embryo but also maintains an undifferentiated cell state in many cellular contexts [16]. Streit *et al *[17] suggested that FGF secreted from a population of organizer precursors initiates induction of neural system before the beginning of gastrulation and Sato *et al *[18] reported that in human embryonic stem cells, *FGF2/13 *expression is enriched and important to understand the molecular mechanisms underlying stemness.

At HH stage 3 (12 h of incubation), three germ layers and germline segregation with the appearance of primordial germ cells (PGCs) begin to form in chick embryogenesis. Since the number of these PGCs is rare at the early developmental stags, it would be difficult to investigate the germline-specific genes. In this study, 12 gene transcripts which were relatively up-regulated in HH stage 3 embryos might be important in germ cell segregation as well as morphological events. Interestingly, Gga.12446.1.S1_s_at, transcript up-regulated at HH stage 3, was *gallus gallus staufen *homolog to *Drosophila STAU1 *and gradually increased during developmental process. *Staufen*, a double-stranded RNA binding protein, was first identified in Drosophila as an essential factor required for germline specification and the anterior-posterior axis formation [19]. Ramasamy *et al*. [20] recently reported that Zebra fish *Staufen1 *and *Staufen2 *are required for the survival and migration of primordial germ cells.

*Septin2 *(GgaAffx.11516.1.S1_s_at), GTP-binding protein, is involved in cytokinesis in various species from yeast and vertebrates [21]. Expression level of *Septin2 *was significantly found in mouse embryonic heart in a developmentally regulated fashion and gradually down-regulated around birth [21]. In our GeneChip data, *Septin2 *were expressed higher at HH stage 3 than at stage X and it might be that embryonic cells actively proliferate and organogenesis begins during gastrulation. However, the results from quantitative RT-PCR showed *septin2 *transcript consistently expressed from stage X to HH stage 9. Thus, spatiotemporal regulation(s) of *septin2 *transcript expression remain to be further investigated.

Although the number of genes analyzed in this study was limited, our data would be contributed to understanding how developmentally important genes are integrated with developmental processes and germline segregation during early embryogenesis in the chicken. Further experiments examining *in situ *cellular expression and functional properties of the identified genes would be needed to elucidate the transcriptional control mechanisms of gene expression associated with early embryogenesis in chickens.

## Conclusion

In this study, we identified a set of genes with stage-specific expression using microarray Genechip and quantitative RT-PCR during the early embryo development in avian species. Discovering stage-specific genes will aid in uncovering the molecular mechanisms involved the formation of the three germ layers and germ cell segregation in the early chick embryos.

## Methods

### Sample preparation and RNA extraction

Experimental animals for this study were maintained at the University Animal Farm, Seoul National University, Korea. All experimental procedures were performed at the affiliated laboratories of the university.

Eggs were brought to the lab within 1 to 3 h of oviposition for stage X embryos [4]. Developing embryos under relative humidity of 60–70% at 37.8°C were staged according to the Hamburger and Hamilton (HH) classification system [6]. Thus, Eyal-Giladi and Kochav (EK) stages X, Hamburg and Hamilton (HH) stage 3, HH stage 6, and HH stage 9 correspond to embryos at 0 and 12 h, 24 h, and 30 h of incubation, respectively.

Total RNA from embryos at each stage was isolated using the TRIzol Reagent (Invitrogen, Carlsbad, CA), according to the manufacturer's instructions. RNA quality was checked by agarose gel electrophoresis and RNA quantity was determined by spectrophotometry at 260 nm. RNA was then further purified using the RNeasy kit (Qiagen, Valencia, CA). The total RNA was used for gene expression analysis on an Affymetrix GeneChip Chicken Genome Array (Affymetrix, Santa Clara, CA), containing 38,535 probes.

### Generation of microarray data

All experiments were performed in triplicate. The generation of GeneChip data from the embryos of stage X and HH stages 3 was performed by Seoulin Bioscience Corporation (Seoul, Korea). Specifically, total RNA (about 5 μg) from stage X and HH stages 3 embryos was used for labelling. Probe synthesis from total RNA samples, hybridization, detection, and scanning were performed according to standard protocols from Affymetrix. Briefly, cDNA was synthesized using the One-Cycle cDNA Synthesis Kit (Affymetrix). Single-stranded (ss) cDNA was synthesized using Superscript II reverse transcriptase and T7-oligo (dT) primers at 42°C for 1 h. Double-stranded (ds) cDNA was obtained using DNA ligase, DNA polymerase I, and RNase H at 16°C for 2 h, followed by T4DNA polymerase at 16°C for 5 min. After cleanup using a Sample Cleanup Module (Affymetrix, Santa Clara, CA), ds cDNA was used for *in vitro *transcription (IVT). cDNA was transcribed using the GeneChip IVT Labeling Kit (Affymetrix) in the presence of biotin-labeled CTP and UTP. Then the biotin-labeled IVT-RNA was fragmented and hybridized to the chicken genome GeneChip array at 45°C for 16 h, according to the manufacturer's instructions. After hybridization, the arrays were washed in a GeneChip Fluidics Station 450 with a non-stringent wash buffer at 25°C, followed by a stringent wash buffer at 50°C. After washing, the arrays were stained with a streptavidin-phycoerythrin complex. After staining, intensities were determined with a GeneChip scanner, controlled by GeneChip Operating Software (GCOS; Affymetrix).

### Microarray data analysis

The quality of the array image was assessed as described in the GeneChip expression analysis manual (Affymetrix). All arrays were processed to determine the "robust multi-array average" (RMA; [22]) using the "affy" software package [23]. Expression values were computed in detail from raw CEL files by applying the RMA model of probe-specific correction for perfect-match probes. These corrected probe values were then subjected to quantile normalization, and a median polish was applied to compute one expression measure from all probe values. Resulting RMA expression values were log_2_-transformed. Clustering from the normalized expression data of all probes was performed and displayed using Cluster and TreeView software [24].

Individual gene expression levels from embryos at stage X and HH stages 3 were compared using an unpaired Student's *t*-test. The Benjamini-Hochberg correction for false discovery rate (FDR) was used for all probe-level normalized data. We selected differentially expressed genes that met the following criteria: a change in expression of at least twofold when comparing the means of the two groups and a FDR-adjusted *P *value of less than 0.01 according to the unpaired Student's *t*-test. Gene Ontology annotation was conducted using NetAffx tool provided by the Affymetrix [25]. Further analysis for functional annotation clustering was performed using the DAVID database [26] [see Additional file 1].

### Quantitative RT-PCR

To confirm the GeneChip expression data and the relative gene expression profiling, quantitative RT-PCR was performed at the developmental stages. Based on microarray data, we selected 36 genes and primers were designed using the Primer 3 software [27] on sequences from the GenBank database (Table 3, 4). For quantitative RT-PCR, we extended embryo development stages from stage X to stage 9 and so prepared total mRNAs from 4 developmental points; stage X (0 h), HH stage 3 (12 h), HH stage 6 (24 h), and HH stage 9 (30 h). sscDNA was synthesized from total RNA (1 μg) using the Superscript III First-Strand Synthesis System (Invitrogen, Carlsbad, CA). The cDNA was serially diluted fivefold and was quantitatively equalized for PCR amplification. Quantitative RT-PCR was performed using the iCycler iQ real-time PCR detection system (Bio-Rad Laboratories, Hercules, CA) and SYBR Green I (Sigma, St. Louis, MO). The glyceraldehyde-3-phosphate dehydrogenase (*GAPDH*) gene was run simultaneously as a control and used for normalization. Non-template control wells with no cDNA were included as negative controls. Each test sample was run in triplicate.

Following the standard curve method, the expression quantities of the examined genes were determined using the standard curves and the Ct values, and were normalized using *GAPDH *expression quantities. The PCR conditions were 94°C for 3 min, followed by 40 cycles at 94°C for 30 sec, 60°C for 30 sec, and 72°C for 30 sec, using a melting curve program (increasing temperature from 55 to 95°C with a heating rate of 0.5°C per 10 sec) and continuous fluorescence measurement. Sequence-specific products were identified by generating a melting curve. The Ct value represents the cycle number at which a fluorescent signal rises statistically above background and the relative quantification of gene expression was analyzed by the2^-ΔΔCt ^method [28]. Based on the quantification RT-PCR results, hierarchical clustering was performed, and the relative quantification of gene expression was normalized to the Ct of stage X as a control group.

## Authors' contributions

BRL carried out all the microarray experiment and quantitative RT-PCR, HK, SC and TP carried out the microarray data analysis, JYH, JML and HK conceived and coordinated the study, and BRL and TSP wrote the manuscript. Figures and quantitative RT-PCR results were prepared by BRL and SM. All authors read and approved the final manuscript.

## Supplementary Material

Additional File 1 Functional annotation clustering of a set of genes identified from microarray experiment. This data represent 5 term clusters generated from functional annotation clustering of total 40 genes identified from microarray results using DAVID database provided by NIAID. Click here for file
